# Metastatic Prostate Adenocarcinoma Posing as Urothelial Carcinoma of the Right Ureter: A Case Report and Literature Review

**DOI:** 10.1155/2014/230852

**Published:** 2014-08-13

**Authors:** Tian-bao Huang, Yang Yan, Huan Liu, Jian-ping Che, Guang-chun Wang, Min Liu, Jun-hua Zheng, Xu-dong Yao

**Affiliations:** ^1^Department of Urology, Shanghai Tenth People's Hospital, Tongji University, 301 Yanchang Road, Shanghai 200072, China; ^2^Department of First Clinical Medical College, Nanjing Medical University, Nanjing, Jiangsu 210029, China

## Abstract

This is a case report of a 67-year-old patient with distant metastasis of prostate cancer to the right ureter which caused hydronephrosis. At the beginning, both of the cytology of the morning urine and imaging findings were consistent with urothelial carcinoma. Nephroureterectomy was subsequently performed. Interestingly, the pathological examination of the excised ureter revealed that the malignancy was derived from the prostate. No skeletal metastasis was detected. However, after four months of follow-up, several abnormal signal shadows were reported in skeletal scintigraphy and the prostate specific antigen (PSA) was gradually increasing. We present such a case for its unique presentation. A review of the literature is also provided.

## 1. Introduction

Prostate cancer is the second most frequently diagnosed cancer and the sixth leading cause of cancer death in men worldwide. About twenty percent of these patients suffer from metastatic disease [1], but ureter involvement is extremely rare. Hydronephrosis, one of the most frequent clinical presentations caused by ureter factors, is mainly due to local extension and external compression by enlarged lymph nodes among patients with cancer. We reported such a rare case presenting with hydronephrosis secondary to ureteral discrete metastasis of prostate adenocarcinoma.

Besides, we searched the Medline database to identify relevant case reports, using the following key words: “prostate” and “cancer or tumor or carcinoma or neoplasm” and “ureteral or ureter” and “metastasis or metastatic” and “case report or review.” In addition, the reference lists of every case report and reviews were also manually searched. As a result, 45 cases of prostate cancer with ureteral metastasis were reported worldwide so far.

## 2. Case Presentation

**Figure 1:**
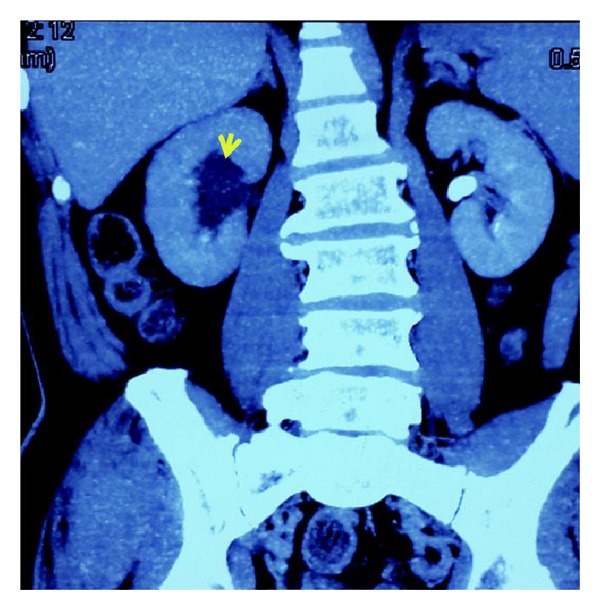
Computed tomography urography demonstrated right hydronephrosis secondary to thickening of the distal ureter.

A 67-year-old man presented with lower urinary tract symptoms and right flank pain for three months. The serum prostate specific antigen (PSA) and free PSA (fPSA) values were 13.07 ng/mL and 0.22 ng/mL, respectively. Digital rectal examination revealed a harder prostate gland. Computed tomography urography (CTU) demonstrated right hydronephrosis secondary to thickening of the distal ureter (Figure 1), mass of the right lateral bladder wall, and benign prostatic hyperplasia (BPH). Enhanced CT scan was conducted subsequently and showed thickening of distal ureter on the right side, with about 6 cm length.

Transrectal ultrasound guided biopsy was conducted firstly and pathological reports showed prostate adenocarcinoma with Gleason score 4 + 5 = 9. Ureteroscopy was performed shortly after mass of bladder was excised and pathologic examination revealed metastases of prostate adenocarcinoma. Immunohistochemical staining of the tumor tissue showed that prostate specific antigens (PSA), P504s and Ki67, were positive, while cytokeratin 7 (CK7), CK20, epidermal growth factor receptor (EGFR), P53, Syn, and Cga were negative. In ureteroscopy, we also found that the mucosa between space occupying focus of bladder and ureteral orifice was normal and free from tumor deposits. However, the ureteroscopy was hard to pass the stricture of the right distal ureter, so it is impossible to obtain the biopsy result. No skeletal or lung metastasis was detected by skeletal scintigraphy and chest radiography, respectively.

**Figure 2:**
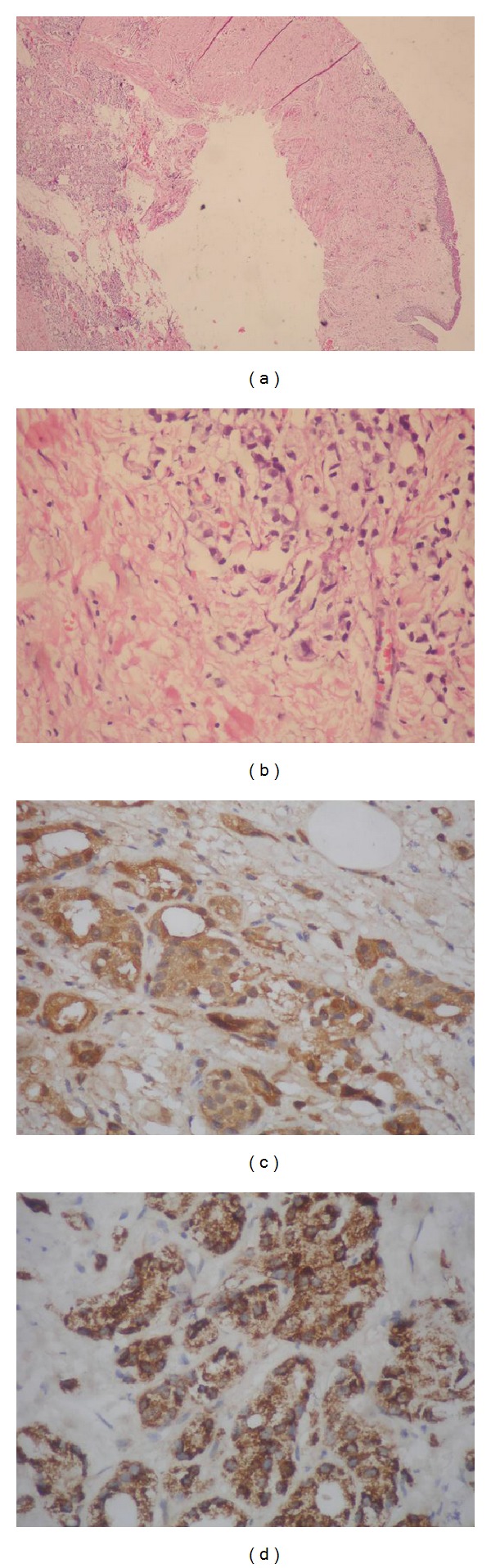
Histopathology of the right ureter represented a skipped lesion of a metastatic prostate adenocarcinoma. ((a), (b)) Histopathology of the right ureter revealed metastatic prostate adenocarcinoma. (a) Histopathology of the ureter representing adenocarcinoma of the prostate (hematoxylin and eosin ×40), (b) with higher magnification (hematoxylin and eosin ×400). ((c), (d)) Excisional biopsy of the right ureteral mass staining for prostate specific antigen (PSA) and P504s in slides (c) and (d), respectively.

Three days' morning urine was collected for cytological examination, one of which revealed urothelial carcinoma. Combining with imaging findings and pathologic report of the bladder masses, a distal ureteral transitional cell carcinoma (TCC) was thought to be most likely. Considering its higher degree of malignancy, nephroureterectomy was recommended. Before removal of the kidney, intraoperative frozen section was submitted and the histology result was in accordance with urine cytology, revealing ureteral TCC. Finally, nephroureterectomy was carried out. To our surprise, immunohistochemical staining of the tumor tissue revealed that the PSA and P504s+ were positive (showed in Figure 2), all of which indicated that the urothelial carcinoma of the ureter turned out to be metastasis of prostate cancer.

The patient reported a significant improvement of lower urinary tract symptoms after nephroureterectomy. The patient was reassessed in two weeks after surgery. His creatinine was slightly increased to 116.4 *μ*mol/L (normal range: 59–104 *μ*mol/L), while the urea nitrogen remained normal. He was treated with luteinizing hormone releasing hormone analogue injection (goserelin Acetate SR Depot) and testosterone and PSA level were monitored every two months. The testosterone was maintained at approximately 0.4 nmol/L after eight months' follow-up. The PSA level decreased to 0.045 ng/mL six months later and then increased at eight months (0.16 ng/mL). Four months later, three skeletal metastases were found in skeletal scintigraphy and pelvic magnetic resonance.

## 3. Discussion

In 1999, a total of 38 cases of ureteral metastases of prostate carcinoma were collected and reviewed by Haddad [2]. Since then, seven new cases were reported in the recent ten years [3–9] (Table 1). Among these cases, chief complaints resulting from ureteral obstruction, such as flank pain and enuresis, account for the main part. For the complete obstruction, urgent transcutaneous nephrostomy was recommended [5]. And for the incomplete obstruction, assessment of renal function and clarifying of the cause of the obstruction should be taken into account firstly. If the hydronephrosis was serious, bilateral double J stents may be also a good choice [6]. Currently, no relevant case with GS < 6 was reported having ureteral metastasis. Therefore, we speculated that high level of GS score (from 3 + 4 to 5 + 5) might be associated with higher risk of ureteral metastasis, compared with GS < 3 + 3.

We presented such a case with lower urinary tract symptoms and right flank pain. Both PSA level and biopsy of the prostate revealed prostate adenocarcinoma. CTU before hospitalization showed a hydronephrosis secondary to ureterostenosis. The biopsy of the thickened ureter is important in the selection of therapeutic measures, but it was hard to be obtained due to the distal obstruction of the ureter. Pathologic examination of bladder masses revealed metastases of prostate adenocarcinoma, which might rule out the possibility of idiopathic bladder cancer. Combined with the urine cytology and imaging findings, ureteral TCC was thought to be most likely. Ureteral surgical exploration was then conducted and the report of the intraoperative frozen section was consistent with ureteral carcinoma. We finally chose to carry out the nephroureterectomy.

It is well known that PSA can be used as the specific marker for prostate cancer. Besides, Molinie et al. demonstrated the ability of P504s to support a diagnosis of prostate cancer, especially, combined with negative staining for a basal cell marker, such as P63 [10]. In addition, a clinical study performed by Liu WH showed that the probability of prostate cancer is 86.36%, when all of the CK7, CK20, and villin were negative. In this case, immunohistochemical staining of tumor tissue from ureter revealed that PSA and P504s were positive, while CK7, CK20, and villin were negative. Combining these kinds of cytological markers, metastatic prostate adenocarcinoma was thought to be most likely.

Hydronephrosis secondary to ureteral stricture might be found early when metastatic prostate carcinoma is involving the ureter. When it comes to this situation, conservative treatment like transcutaneous nephrostomy or double J stent insertion may be selected in emergency. We may take benign lesion like stone, BPH, and congenital stricture ureter into account firstly, especially when there was no evidence initially suggesting prostatic disease as the cause. But from this case, we should consider the possibility of metastatic prostate cancer when any lesions in the ureter existing are believed to have a malignant origin.

## 4. Conclusion

The urologist should notice this atypical cause for hydronephrosis secondary to ureteral stricture which prevents passage of ureteroscopy, making it impossible to obtain biopsy result in a patient with carcinoma of the prostate. Besides, cytology of morning urine and intraoperative frozen section may be useful but can never replace pathology due to the existence of a certain degree of error.

## Figures and Tables

**Table 1:** Characteristics of relevant case reports published from January 2000 to November 2013.

Author	Year	Country	Detailed information of each case report							
			Age	Chief complaint	Hydronephrosis∗	PSA	DRE	Ureter metastasis^Φ^	Treatment	Follow-up
Jallad S	2012	UK	76	Right loin pain	Right (CT)	Nor	Nor	Histology of right NU GS grade (5 + 5)	**Initial:** right ureteroscopy **Secondary:** open exploration and frozen section **Third:** right NU	GnRHa injection^&^

Schneider S	2012	Germany	74	Right flank pain and LUTS	Right (ultrasound and intravenous pyelography)	52	NG	Ureterorenoscopy with biopsy GS grade (3 + 4)	**Initial:** LHRH-analogues **Secondary:** ureterorenoscopy with biopsy **Third:** nephrostomy **Fourth:** LHRH analogues + zoledronic acid infusions once a month **Fifth:**LHRH-analogues and antiandrogene	Died in ALF, which was due to liver metastasis with highly progressive disease

Chalasani V	2010	Canada	68	Right upper quadrant pain	Right (CT)	96	Enlarged, hard prostate	Histology of right NU GS grade (4 + 3)	**Initial:** ADT **Secondary:** NU	NG

Singh G	2009	Singapore	70	Episode of urinary retention	Bilateral (CT)	30	NG	Histology of tumor masses obstructing the ureteric orifices GS grade (4 + 4)	**Initial:** TURP + bilateral orchidectomy **Secondary:** insertion of bilateral double J stents (successful at last)	Obstruction relieved and PSA decreased to 0.16 ug/L

Marzi M^#^	2007	Italy	64	12-hour enuresis	No	/	/	Histology of whitish tissue in ureter	**Initial:** ADT **Secondary:** urgent right transcutaneous nephrostomy	/

Siddiqui E^#^	2004	Japan	69	/	/	19.6	/	Histology	Chemoendocrine therapy	NG

Jung JY	2000	Korea	64	LUTS and gross hematuria	Right (excretory urography)	40.9	NG	Histology of right NU	**Initial:** bilateral orchidectomy **Secondary:** TURP + right percutaneous nephrostomy **Third:** right NU	Three months later, PSA had decreased to 1.3 ng/mL

*‘‘Hydronephrosis” refers to ‘‘if there exists hydronephrosis? Which side? And which exam revealed?”.

^Φ^‘‘Ureter metastasis” refers to ‘‘which examination indicates distant metastases of prostate adenocarcinoma to the ureter?”.

NU: nephroureterectomy, PSA: prostate specific antigen, DRE: digital rectal examination, Nor: normal, ALF: acute liver failure, LUTS: lower urinary tract symptoms, ADT: androgen deprivation therapy, TURP: transurethral resection of the prostate, CT: computed tomography, GS: Gleason grade, GnRHa: gonadotropin-releasing hormone analogue, and NG: not given.

^&^Four weeks later, both alkaline phosphatase and bone scan revealed widespread bony metastasis, and GnRHa injection was used.

^#^ Only abstract available and only part of information displayed in the table.
